# CD4^+^ T Lymphocytes—From Their Multiple Immunological Roles to New Sensing Strategies Using Marine Magnetotactic Bacteria—A Systematic Review

**DOI:** 10.3390/s26113324

**Published:** 2026-05-23

**Authors:** Natalia Lorela Paul, Catalin Ovidiu Popa, Rodica Elena Ionescu

**Affiliations:** 1 Laboratory Light, Nanomaterials and Nanotechnologies—L2n, University of Technology of Troyes & CNRS UMR 7076, 12 Rue Marie Curie, CS 42060, CEDEX, 10004 Troyes, France; 2 Materials Science and Engineering Department, Faculty of Materials and Environmental Engineering, Technical University of Cluj-Napoca, 400641 Cluj-Napoca, Romania; 3 Eut+ Institute for Nanomaterials & Nanotechnologies EUTINN, European University of Technology, European Union

**Keywords:** CD4^+^ T lymphocytes, CD4^+^ immunological roles, CD4^+^-associated pathologies, magnetotactic bacteria, biosensing strategies, microfluidic devices

## Abstract

**Highlights:**

CD4^+^ T cells as central regulators of immune homeostasis, and magnetotactic bacteria in the detection of white blood cells.

**What are the main findings?**

- Current methods are accurate but limited by laborious laboratory infrastructure.
- MTBs as an innovative alternative for CD4^+^ detection.

**What are the implications of the main findings?**

- Possibility of developing more flexible and portable sensing platforms.
- Need for experimentation, standardization and validation for clinical application.

**Abstract:**

CD4^+^ T lymphocytes are important regulators of immune homeostasis, and their dysregulation is closely linked to a wide range of diseases. For this reason, their reliable detection remains a major challenge. Despite the fact that current methods are analytically robust, they rely mainly on laboratory infrastructure, limiting their flexibility and wider applicability. The present review analyzes established and emerging approaches for CD4^+^ T-cell detection, with a focus on their practical limitations regarding portability, flexibility and applicability. To the best of our knowledge, for the first time, the authors are examining the possibility of exploring marine magnetotactic bacteria (MTBs) as active biological elements in immune cell detection due to their intrinsic magnetic properties, biological organization, and surface biofunctionalization potential. Rather than offering an immediate technological solution, the use of MTBs serves as a challenging biological framework that could provide more adaptable and sensitive detection strategies. At the same time, the limitations of this concept are acknowledged, emphasizing the need for further experimental validation, considering that this strategy, although promising, remains an exploratory concept.

## 1. Introduction

The immune system (IS) is the body’s defense mechanism against pathogens and cancer [1]. It is one of the most complex biological networks, with some of the main roles in protection against pathogens, elimination of damaged cells, tolerance of self-structures, and recognition and differentiation of non-self-structures. It is made up of numerous elements and structures and generates a variety of cells and recognition molecules, acting through them to generate an immune response. The proper functioning of the IS is based on the balance between defense reactions and regulatory mechanisms.

From a functional point of view, the IS presents two important and interrelated components: innate and adaptive immunity [1,2]. Innate immunity, also known as nonspecific or natural immunity, is the first line of defense of the body that acts in the early phase of the immune response (IR). In contrast, adaptive, acquired or specific immunity is defined by a high degree of specificity and immunological memory through which it recognizes and selectively eliminates antigens. At the center of this branch, which includes antigen specificity and immunological memory, are T and B lymphocytes.

T lymphocytes are produced in the bone marrow and mature in the thymus, where they undergo rigorous selection processes that ensure the recognition of foreign antigens, without reacting against self-tissues. Depending on the surface markers and the functions exercised, T lymphocytes are classified into several subpopulations, of which CD4^+^ T lymphocytes, also known as T helper cells, play an extremely important role in coordinating the immune response.

CD4^+^ T lymphocytes recognize antigens presented by antigen-presenting cells via major histocompatibility complex (MHC) class II molecules and, once activated, secrete cytokines that influence the behavior of other immune cells [3,4,5]. They function as “conductors” of the immune response, regulating its intensity and direction. Depending on the cytokine environment and the type of antigenic stimulus, CD4^+^ T cells can differentiate into distinct subpopulations, such as Th1, Th2, Th17, Th22, Th9, Tfh, or Treg, each with specific roles in anti-infective defense, inflammation or immune tolerance [6,7,8,9]. This functional plasticity makes CD4^+^ T lymphocytes extremely important, but at the same time, it also makes them vulnerable in pathological contexts.

Maintaining normal CD4^+^ T-cell function is essential for the health of the body, and their number is also important. For instance, a significant decrease in CD4^+^ T cells, as observed, for example, in HIV infection, leads to severe immunosuppression and increased susceptibility to opportunistic infections [10,11]. On the other hand, excessive and/or unbalanced activation of T-cell subpopulations is often associated with autoimmune diseases, allergies or chronic inflammation [9,12,13,14,15]. Beyond infectious diseases, monitoring CD4^+^ T-cell dynamics is increasingly relevant in cancer immunotherapy, autoimmune disorders and immune reconstitution following transplants [16,17]. For this reason, CD4^+^ T cells are considered both key biomarkers for the state of the immune system and important targets for monitoring and therapeutic intervention. Thus, the detection and quantification of CD4^+^ T lymphocytes, with as much accuracy as possible, is of major importance not only in diagnostics but also in basic research and the development of advanced biomedical technologies. Understanding their role in the immune network provides the necessary basis for the design of innovative detection systems, including platforms based on magnetotactic bacteria, capable of exploiting their biological and physical properties for sensitive and selective applications.

Classic methods for analyzing CD4^+^ lymphocytes, such as flow cytometry, are extremely accurate but come with several limitations related to cost, complexity, and lack of portability [18]. Lately, research has been focused on the development of alternative detection platforms capable of providing rapid, selective responses and, ideally, being able to be integrated into miniaturized systems. In this context, biosensors based on optical, electrical or acoustic signals have attracted considerable interest, especially when combined with efficient surface biofunctionalization strategies for the selective capture of target cells.

**Figure 1:**
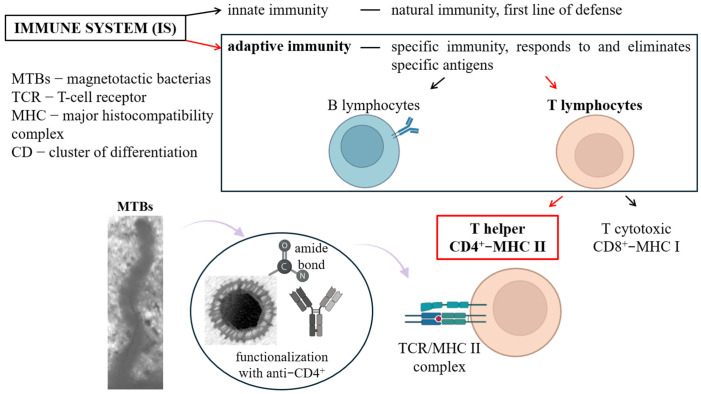
Conceptual framework of CD4^+^ T-cell detection using magnetotactic bacteria; MTBs—magnetotactic bacteria, TCR—T-cell receptor, MHC—major histocompatibility complex, CD4^+^—cluster of differentiation.

An emerging and yet unexplored field is the use of magnetotactic bacteria (MTBs) as functional elements in biosensors for cellular detection. These bacteria are aquatic microorganisms that synthetize magnetosomes, which are well-ordered magnetic nanoparticles that allow them to respond to external magnetic fields [19,20]. Their specific properties, such as biocompatibility, self-organization, and controllable response to magnetic stimuli, make them very attractive biological platforms for biomedical applications, including the separation and detection of immune cells [20,21]. This is relevant for CD4^+^ T lymphocytes, whose surface markers and functional importance make them suitable targets for MTB-based capture strategies. Additionally, the surface of MTBs can be biofunctionalized with antibodies, aptamers or other specific recognition molecules that could allow selective interaction with surface markers of T lymphocytes [19,20,22]. In this way, MTBs could act both as a capture element and as an indirect signal transducer. Consequently, they could be integrated into magnetic, electrochemical or hybrid biosensors. This approach may open the possibility of developing label-free detection systems with precise magnetic manipulation and high potential for point-of-care (POC) applications (see Figure 1).

Despite the significant progress reported in the field of biosensors for the detection of CD4^+^ T lymphocytes, the current literature seems somehow fragmented, and existing studies most often treat immunological aspects, biofunctionalization strategies and types of transductions separately, without coherently analyzing the potential of MTBs as integrated platforms for the selective capture and detection of these cells.

In this review, we aim to analyze the current state of the art in CD4^+^ T-cell detection methods, with a focus on biosensor-based approaches and the potential of using MTBs as biofunctionalized platforms for the selective capture and detection of these cells. In this study, we proposed answers to some specific scientific questions considered relevant in this particular case:

(Q1) How can the biological and magnetic properties of MTBs exploited to improve the sensitivity, specificity and portability of CD4^+^ detection systems, compared to conventional methods currently used?
(Q2) What are the advantages and limitations of different types of transductions (optical, electrical or acoustic) when integrated with biofunctionalization strategies based on MTBs?

To the best of our knowledge, there are currently no dedicated review articles addressing the use of MTBs for CD4^+^ T-lymphocyte detection. The existing literature is generally divided into two separate research directions: (i) well-established documents focusing on CD4^+^ T-cell detection technologies, and (ii) papers addressing the biomedical applications of magnetotactic bacteria and magnetosomes, particularly in drug delivery, imaging, and biosensing. In this context, the present work integrates these two areas and proposes a conceptual framework for the potential use of MTB-based systems in immunological cell detection, with a specific focus on CD4^+^ T lymphocytes.

The novelty of this review lies in the integrative approach that combines CD4^+^ T-cell immunology with MTB biotechnology, providing an interdisciplinary perspective on the development of a new and innovative sensing platform with real potential for biomedical and point-of-care applications. The review first outlines the immunological relevance of CD4^+^ T lymphocytes, then discusses current detection strategies, and finally highlights the potential of MTB-based platforms for future CD4^+^ detection technologies.

## 2. Research Methodology

In order to perform a comprehensive review of the literature based on CD4^+^ T-lymphocyte detection (with a focus on biosensors), specifically the use of magnetotactic bacteria as biofunctionalized platforms, we chose to use a systematic approach based on PRISMA (Preferred Reporting Items for Systematic reviews and Meta-Analyses) 2020 guidelines. The choice for this methodology was motivated by the need to structure the process of identification, selection and inclusion of studies in a reproducible and transparent way. A detailed PRISMA checklist is provided in the Supplementary Materials in Table S1.

In the identification phase, the main database chosen was the Web of Science Core Collection, due to its rigorous indexing criteria, thus ensuring the identification of a central core of relevant articles. To extend the coverage and not omit important contributions, complementary searches were also performed in other databases (mainly Scopus, ScienceDirect and Google Scholar). The latter was used as a complementary source to identify potentially relevant publications that are not indexed in Web of Science.

Querying the database according to the central topic of the paper (CD4^+^ T lymphocytes) yielded 68,835 articles. The keywords we used targeted three main areas: CD4^+^ T lymphocytes (“CD4^+^ T cells”, “T helper lymphocytes”), biosensors and detection technologies (“biosensor”, “detection”, “label-free detection”) and magnetotactic bacteria (“magnetotactic bacteria”, “magnetosomes”, “biofunctionalization”).

The second step was screening. This stage involved narrowing the period of interest to 2022–2025, the language of publication (English), and the type of publication (original articles and reviews, open access). The searches were refined with additional keywords, such as “T cell subsets,” “biofunctionalization,” and “magnetotactic bacteria or magnetosomes,” resulting in 2710 articles eligible for more detailed analysis. Additionally in this stage, we performed a brief screening of titles and abstracts to eliminate irrelevant studies and narrow down the search. Screening was performed independently by reviewing titles and abstracts, followed by full-text evaluation of potentially relevant studies.

**Figure 2:**
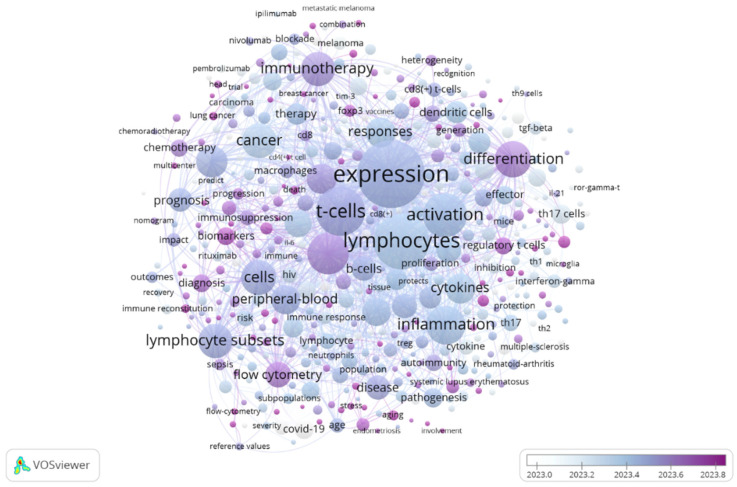
Co-occurrence map of keywords related to CD4^+^ T lymphocytes. Node size reflects the frequency of keyword occurrence, while link thickness indicates the relationship strength between terms. Colors represent different thematic clusters identified within the analyzed literature (for the time period of 2022–2025). The scale bar corresponds to the relative co-occurrence strength between keywords.

In order to analyze thematic trends and relationships in this large body of literature, VOSviewer software was used (version 1.6.20), which allowed the visualization of keyword co-occurrence networks (Figure 2). We chose a co-occurrence threshold of 10, given the large number of articles identified, to narrow the spectrum to the central concepts relevant to the topic of the article. The analysis highlighted several main thematic clusters based on the 444 keywords (out of a total of 9656).

One of the clusters is focused on the cellular expression and characterization of T lymphocyte subpopulations, with a focus on CD4^+^ cells. The central cluster, dominated by terms such as “expression”, “lymphocytes” and “subsets”, suggests an interest in identifying specific surface markers and the functional differentiation of these cells. This direction is highly relevant for the development of detection methods since any protocol for the biofunctionalization of a support must consider the particularities of CD4^+^ expression and its variations under physiological or pathological conditions.

Another thematic trend links CD4^+^ cells to immune responses, cytokines and cellular differentiation processes. The emergence of terms such as “cytokines”, “T helper cells” and “Th17 cells” may suggest that CD4^+^ T cells are not viewed as simple marker cells but as key elements in maintaining the balance of the immune system. Imbalances in these subpopulations are frequently associated with autoimmune diseases, chronic inflammation or persistent infections, which justifies the need for sensitive detection systems capable of distinguishing between normal ranges and states of immunological abnormality.

The cluster associated with pathology, including cancer, infectious diseases and inflammation, tells us that CD4^+^ T cells are frequently used as biomarkers in diverse clinical contexts. In this sense, the cluster analysis supports the idea that lymphocyte biosensors need to be adapted for real-world applications, where cell concentrations are variable and where biological interference is unavoidable. The integration of magnetotactic bacteria into such systems appears to be a promising solution, especially due to their natural magnetic properties, which can be exploited for separation, guidance and amplification of the detection signal. In addition, there is a methodological cluster in which terms such as “flow cytometry”, “peripheral blood” and “biomarkers” appear. It reflects the classical methods of lymphocyte analysis, which serve as a reference for the development of modern biosensors. Compared to these standard techniques, optical, electrical and acoustic biosensors offer the advantage of faster and, potentially, portable detection. The cluster analysis may suggest that the current direction of research is towards combining these methods with advanced biofunctionalized platforms in order to achieve high specificity towards CD4^+^ lymphocytes. Thus, the structure of the cluster suggests a clear convergence between fundamental immunology, Th lymphocyte characterization and the development of innovative biosensors. All of it supports the multidisciplinary approach of the present work in which the classification of T cells, the analysis of their role in maintaining immune homeostasis and the development of detection systems based on biofunctionalized supports are closely interconnected and complement each other.

**Figure 3:**
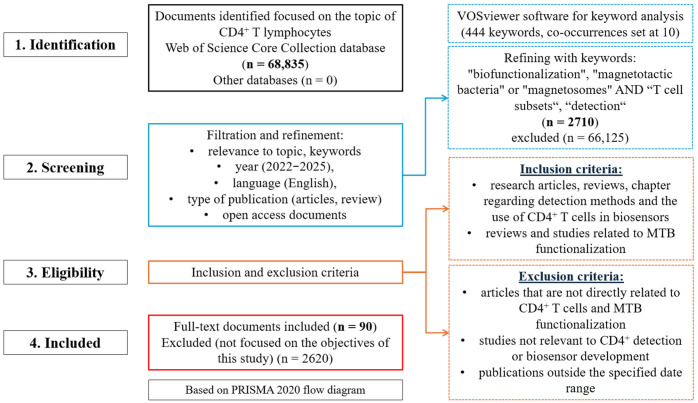
Literature research methodology on T-cell detection methods and MTB functionalization; MTBs—magnetotactic bacteria.

After the thematic trend analysis, we continued with the eligibility stage in which articles were evaluated depending on the application of clear inclusion and exclusion criteria. Studies addressing Th-cell detection methods, biosensor platforms, and in particular the use of MTBs as biofunctionalized elements were included. Papers that did not directly focus on Th cells or MTBs, studies that did not present methodological details relevant to biosensors and publications outside the specified period were excluded. Finally, 90 articles were selected for detailed analysis. We refer to Figure 3 for a schematic representation of the bibliographic research methodology.

Data extracted from the articles included the type of study, the detection principles used, the type of biosensor, biofunctionalization strategies of magnetotactic bacteria and performance indicators (sensitivity, specificity). Instead of a quantitative meta-analysis, the data were qualitatively synthesized to identify trends, technological gaps and emerging opportunities. A quantitative meta-analysis was not performed due to the heterogeneity of detection platforms, transduction mechanisms and experimental designs across the included studies.

This systematic approach, based on PRISMA and complemented by VOSviewer co-occurrence network analysis, provided a clear, transparent and reproducible methodological framework that highlights both the current state of the field and the innovative directions proposed for CD4 T-cell detection with biofunctionalized platforms based on magnetotactic bacteria.

The novelty of the methodological part of this review lies in the integrative use of PRISMA 2020 guidelines and bibliometric network analysis, where VOSviewer is employed not only as a descriptive tool but also as a conceptual filter to refine and structure the literature. By applying a high co-occurrence threshold, this approach let us focus on the identification of core research themes at the intersection of CD4^+^ T-cell immunology, biosensor technologies and magnetotactic bacteria, thereby reducing thematic dispersion and highlighting underexplored interdisciplinary connections.

## 3. Results

### 3.1. T Lymphocytes—Central Regulators of Immune Homeostasis

T lymphocytes are one of the most important cellular components of the adaptive immune system [23]; they act as central regulators of homeostasis and immunological responses. In contrast to innate immune cells, which respond rapidly and nonspecifically to pathogens, T lymphocytes are characterized by high antigen specificity, clonal expansion, and immunological memory [24,25]. The aforementioned characteristics give T cells not only a crucial role in long-term protection against infections but also make them relevant biological indicators of the state of the immune system. Specificity and immune dynamics make these immune cells valuable for advanced detection technologies, including biosensors.

T cells, which originate from hematopoietic stem cells in the bone marrow, mature in the thymus. During their development, they undergo a series of selection processes [26,27]. These processes eliminate cells that either do not recognize MHC molecules or react too strongly to self-antigens.

**Figure 4:**
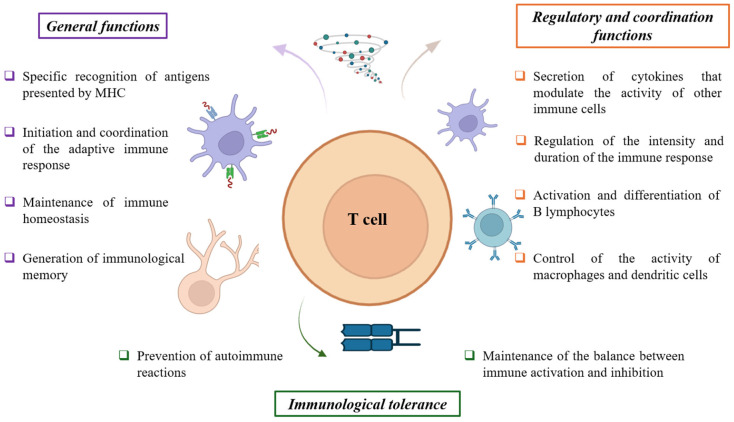
Essential roles and functions of T lymphocytes.

T lymphocyte roles in the immune system go beyond simple antigen recognition [28,29,30,31] (see Figure 4). They act as veritable command centers that coordinate, amplify, or limit immune responses. Through direct interactions with other immune cells and through the secretion of cytokines, T lymphocytes influence both the intensity and direction of the immune response, ensuring a fine balance between protection and tolerance. After the recognition of an antigen, activated T cells initiate a series of intracellular cascades that lead to proliferation and functional differentiation [32,33]. This tightly regulated process is dependent on multiple signals, including costimulation and the local cytokine environment. The controlled activation of T lymphocytes is important for an efficient immune response, while aberrant or insufficient activation can lead to significant immunological disorders.

T lymphocytes specifically recognize antigens and initiate the adaptive immune response. Through the T-cell receptor (TCR), they can identify antigenic fragments presented by MHC molecules, which allows them to distinguish self-structures from non-self-structures [34,35]. This specific recognition capacity is the basis of the directed immune response and is a key element in protecting the body against infections and malignant cellular transformations.

Classified based on the expression of surface markers and their functional role, the two major subpopulations of lymphocytes are CD4^+^ and CD8^+^ T cells, each with distinct roles in immunity [36]. CD4^+^ T lymphocytes, known as helper cells, coordinate the immune response through cytokine secretion and interactions with other immune cells, while CD8^+^ T lymphocytes are primarily involved in direct cytotoxic activity against infected or malignantly transformed cells [33,36]. The distinction between the two categories is of major importance, especially when developing detection strategies that allow specific differentiation of the subpopulations.

CD4^+^ T cells play a main role in the orchestration of the intracellular cascades, acting as mediators between innate and adaptive immunity [23,37,38,39,40]. Through the secretion of specific cytokines, they modulate the activity of macrophages, B lymphocytes and CD8^+^ T lymphocytes, influencing antibody production, cytotoxic activity and the maintenance of immunological memory. Thus, CD4^+^ T cells are not only effector cells but also regulators of the entire immune ecosystem.

A particularity of T-cell function is their ability to rapidly adapt to the biological context [41,42,43]. Depending on the type of antigen, the location of infection and inflammatory signals, T lymphocytes respond through the profile of secreted cytokines and cellular interactions. This functional plasticity allows the immune system to respond effectively to a wide range of threats, but at the same time, it represents a source of vulnerability. In addition, T cells are actively involved in maintaining immunological tolerance [44,45,46]. Negative regulatory mechanisms, including the suppression of excessive responses and the elimination of autoreactive cells, contribute to preventing immune attack against self-tissues. This role is all the more important in the context of autoimmune diseases, where the imbalance between the activation and regulation of the immune response is a central element of pathogenesis. These complex functions make T cells not just passive participants in the immune response but also dynamic indicators of the overall immunological state.

### 3.2. T-Lymphocyte Subpopulations and Functional Plasticity

T lymphocytes are not a homogeneous cell population, but rather, a highly diverse group, characterized by significant differences in phenotype, function, and biological behavior. This functional diversity is imperative for the ability of the immune system to respond effectively to a wide variety of stimuli, from acute infections to chronic inflammatory processes.

Based on the expression of surface markers, T cells are categorized as (i) CD8^+^ T lymphocytes and (ii) CD4^+^ T lymphocytes. CD8^+^ T lymphocytes are known for their cytotoxic role and are involved in the direct elimination of virally infected cells or tumor cells [47,48,49]. They act through mechanisms such as the release of perforin and granzymes, inducing apoptosis of target cells. In contrast, CD4^+^ T cells play a role in coordinating and regulating immune responses. They act through cytokine secretion and interactions with other immune cells, influencing both innate and adaptive immunity. Their importance is underscored by the fact that dysregulation of CD4 populations is associated with a wide range of pathologies, from severe infections to autoimmune diseases and cancer [9,23,31,50].

A defining aspect of CD4^+^ T lymphocytes is their functional plasticity. Depending on the signals received upon activation, particularly the cytokine involved and the type of antigen, these cells can differentiate into several distinct subpopulations, each characterized by a specific cytokine profile and biological functions [33]. This adaptive capacity allows for a personalized immune response but also introduces a high level of biological complexity.

**Figure 5:**
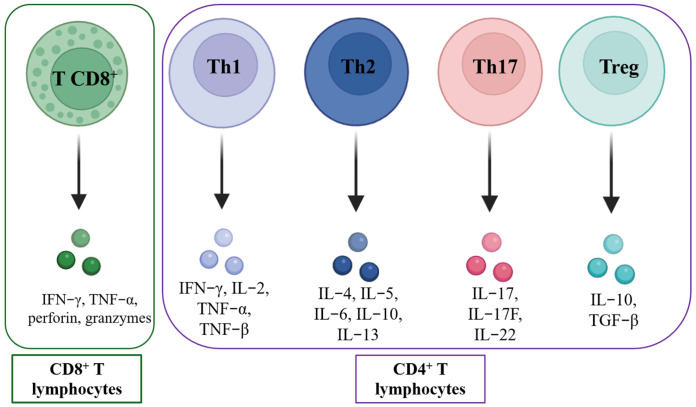
Schematic representation of the main categories of lymphocytes and the main cytokines secreted.

Several types of CD4^+^ T-cell subsets have been described in the literature, and in this section, we will mention some of them along with some of their defining characteristics. In Figure 5, we schematized the main categories of T cells and the cytokines secreted by them.

**Th1 lymphocytes** are predominantly involved in immune responses against intracellular pathogens, such as bacteria and viruses. They mainly secrete interferon-gamma (IFN-γ), tumor necrosis factor α and β (TNF-α, TNF-β), and IL-2 and IL-12, which are cytokines that activate macrophages and support the cytotoxic response of CD8^+^ T lymphocytes [33,51,52]. An efficient Th1 response is essential for the control of viral infections, but excessive activation can contribute to tissue inflammation and autoimmune diseases.

**Th2 lymphocytes** are associated with immune responses against extracellular parasites and allergic reactions. They secrete cytokines such as IL-4, IL-5, IL-6, IL-10, and IL-13, which stimulate B-cell activity and the production of IgE antibodies. Although Th2 responses are important for defense against certain pathogens, their dysregulation is frequently implicated in allergies and asthma [53,54,55,56].

**Th17 lymphocytes** have a role in the defense against extracellular bacteria and fungi, especially at the epithelial barrier level, and a role in the clearance of pathogens that are not adequately handled by Th1 or Th2 lymphocytes [57,58,59]. They are characterized by the secretion of IL-17, IL-17F and IL-22, which are cytokines that induce local inflammation and neutrophil recruitment. Although this response is essential for protection against infections, uncontrolled activation of Th17 lymphocytes is associated with chronic inflammation and autoimmune diseases [60,61,62,63].

**Regulatory T lymphocytes** (Tregs) play a role in limiting excessive immune responses and maintaining immunological tolerance. They exert their function mainly through the secretion of anti-inflammatory cytokines, such as IL-10, IL-35 and TGF-β, as well as through cytokine consumption mechanisms (IL-2) [64]. They suppress the activity of other immune cells by secreting proinflammatory cytokines such as IL-6 or TNF-α. Dysfunctions of Treg populations are associated with the development of autoimmune diseases and persistent inflammation, while their excessive activity may contribute to tumor evasion [64,65,66].

In Table 1, we have summarized the main T-cell populations, highlighting the surface markers, cytokine profiles (or their effector molecules), and dominant functional roles of each subpopulation, as well as the types of immune response in which they are involved, providing a useful framework for understanding the functional complexity of T lymphocytes and their relevance as targets for the development of advanced detection systems.

#### Multi-Marker Profiling of Th-Lymphocyte Subpopulations

Although the CD4 marker is commonly used to detect T helper cells, it is important to acknowledge the heterogeneity of these populations. The latter cannot be adequately characterized by analyzing just a single surface marker. Their functional diversity is often defined by the co-expression of additional markers. These markers allow for the differentiation of subpopulations involved in immune regulation, activation, and memory.

An example of such heterogeneity refers to regulatory T cells. Tregs [69,70] are frequently characterized by the co-expression of CD4^+^CD25^+^ markers, particularly accompanied by the expression of the transcription factor FOXP3. In this form, they participate in maintaining immune tolerance and limiting excessive inflammatory responses. The co-expression of the CD25 and FOXP3 markers is considered definitive for the precise identification and differentiation of Tregs from other activated helper subtypes.

Another population of interest is that of double-positive (DP) CD4^+^CD8^+^ T lymphocytes [71,72,73]. Initially, these cells were considered to be exclusively immature forms present in the thymus; however, research has demonstrated their presence in peripheral blood and lymphoid tissues. These subpopulations exhibit high functional heterogeneity and can display helper, cytotoxic, or regulatory properties [74]. An increase in CD4^+^CD8^+^ populations has been associated with inflammatory conditions, chronic viral or parasitic infections, autoimmune diseases, and cancer [73].

In addition to these markers, there are other surface molecules. Among them, we mention CD45RA [75], CD45RO [76], CCR6 [77], CXCR3 [78], CCR4 [79], and PD-1 [80,81]. Their presence and expression are often used to distinguish between naive, effector, memory, and functionally exhausted subsets in various inflammatory conditions, sepsis, and other alterations in the immune response. These combinations of markers allow for a much more precise characterization of the immunological status and biological function of lymphocyte populations. The assessment of these markers plays a particular role in studying their dynamics under various conditions or pathologies.

The simultaneous determination of multiple CD markers has become an essential step in modern immunology and in the development of advanced cell detection platforms. Multiparametric analysis enables the identification of complex immune signatures and the differentiation of closely related subsets that cannot be distinguished using single-marker-based methods. For this reason, modern technologies are increasingly used for high-resolution immunological profiling and for the early monitoring of inflammatory, autoimmune, and oncological diseases.

### 3.3. CD4 Population Abnormalities and Associated Pathologies

**Figure 6:**
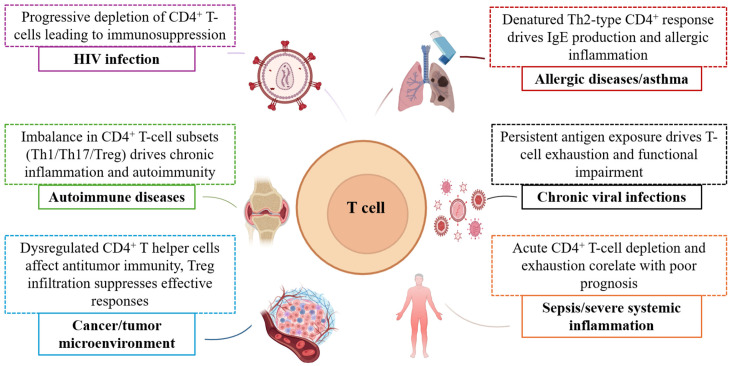
Examples of pathologies associated with CD4^+^ T-cell abnormalities: HIV infection [82,83,84,85,86], autoimmune disease [13,87,88,89,90,91], cancer [92,93,94,95], allergic diseases [96,97,98,99], chronic infections [100,101,102,103,104], and systemic inflammation [105,106,107,108,109].

As can be seen from the previous classification, we can say with certainty that CD4^+^ T lymphocytes are essential for maintaining immunological balance. However, this balance is delicate and often easily perturbed. Changes in the number, distribution or function of CD4^+^ subpopulations can reflect or even determine the emergence and evolution of pathological conditions. Understanding these disorders is vital not only for immunology but also for the development of detection systems capable of distinguishing between physiological and pathological states. In Figure 6, we have summarized some of the main pathologies associated with CD4^+^ T-cell abnormalities.

#### 3.3.1. HIV Infection

One of the best-known and best-documented situations in which CD4^+^ T-cell populations are significantly disrupted is infection with HIV [82,83,84,85,86]. The latter specifically infects CD4^+^ lymphocytes and uses them as a host for replication; this leads to a progressive decrease in the number of CD4 cells in the blood. This decrease causes severe immunosuppression, which makes the body vulnerable to opportunistic infections and malignancies. Monitoring the number of CD4^+^ T cells has thus become a standard clinical indicator for assessing HIV progression and the effectiveness of antiretroviral therapy.

#### 3.3.2. Autoimmune Diseases

Infections are not the only conditions that alter CD4^+^ populations. In autoimmune diseases, such as rheumatoid arthritis, systemic lupus erythematosus, multiple sclerosis, inflammatory bowel disease (IBD), and Crohn’s disease, there is often dysfunction of CD4^+^ subpopulations, especially regulatory T cells (Tregs) and Th17 subsets [13,87,88,89,90,91]. An imbalance in the Th17/Treg ratio is associated with the immune system’s tendency to generate excessive proinflammatory responses that target self-tissues, leading to chronic inflammation and tissue damage. This reflects not only an altered number of CD4^+^ cells but also a dysfunctional profile, which has direct implications for the diagnosis and management of autoimmune diseases.

#### 3.3.3. Cancer Diseases

In the context of cancer, one of the most aggressive and insidious diseases, CD4^+^ populations may also have an ambivalent role. In some situations, an increased number of active CD4^+^ T lymphocytes involved in the antitumor response may contribute to the recognition and destruction of malignant cells. In other situations, certain subsets, such as Tregs, may promote immune tolerance towards tumor cells, allowing them to evade immune surveillance [92,93,94,95]. This duality makes the analysis of CD4^+^ populations relevant not only quantitatively but also qualitatively when assessing prognosis and the response to immunotherapy in cancer.

#### 3.3.4. Allergic Diseases

Another relevant example is that of allergic diseases and asthma, where the predominance of Th2 subsets, with increased secretion of IL-4, IL-5 and IL-13, contributes to unbalanced immune responses based on exaggerated IgE production and eosinophil involvement, leading to characteristic clinical symptoms [96,97,98,99]. These pathological models clearly show that examining CD4^+^ populations can provide important information about disease mechanisms and guide appropriate therapeutic interventions.

#### 3.3.5. Chronic Infections

Chronic inflammation, whether caused by persistent infections or metabolic processes, also affects CD4^+^ populations [100,101,102,103,104]. In diseases such as type 2 diabetes, metabolic syndrome, and cardiovascular disease, a perturbed immune response, in which CD4^+^ subpopulations play an active role, contributes to the maintenance of the inflammatory state. In this case, it is not necessarily a simple numerical decrease or increase, but rather, a change in the functional and cytokine profiles of these cells.

#### 3.3.6. Systemic Inflammation

Immune disorders caused by CD4^+^ imbalances are not limited to classical immunological conditions. In severe bacterial infections, such as sepsis, there is often a profound decrease in T-lymphocyte counts, which is correlated with an unfavorable, or one might even say serious, prognosis [110,111,112,113,114]. Similarly, in acute viral infections, such as COVID-19, disruptions of CD4^+^ populations have been mentioned to be associated with disease severity and generalized immune dysfunction [105,106,107,108,109].

Dysregulation of CD4^+^ T-lymphocyte populations is present in a wide range of pathologies, from viral and bacterial infections to autoimmune diseases, cancer, chronic inflammation and metabolic disorders. These changes are not limited to numerical variations but involve profound changes in the distribution of subpopulations and in the functional or cytokine profiles of CD4^+^ cells. Therefore, the assessment of the state of the immune system requires detection methods capable of capturing both the quantitative and qualitative aspects of these populations. In this context, CD4^+^ T lymphocytes are essential biomarkers for diagnosis, monitoring and prognosis, which raises the need to develop advanced detection technologies that are sensitive and adaptable to diverse clinical contexts.

### 3.4. Current Methods Used for CD4^+^ T-Lymphocyte Detection

The detection and quantification of CD4^+^ T lymphocytes is a central step in both clinical practice and basic research. This is all the more relevant in the context of the central position of these cells in maintaining immune homeostasis. Over time, several established methods have been developed for the identification and analysis of CD4^+^ T cells, each with clear advantages but also with limitations that are becoming increasingly evident in the context of the need for rapid, portable and accessible diagnostics. In Table 2, we have summarized a couple of the methods used for T-lymphocyte detection along with their advantages, limitations, and applications.

Currently, there is a relatively wide range of methods used to identify and quantify CD4^+^ T cells. However, the vast majority of them have been designed and developed in a controlled laboratory context, with an emphasis on analytical accuracy rather than flexibility or portability. This reality creates some tension between methodological performance and practical applicability, which becomes increasingly evident when we consider the current needs in diagnostics, real-time monitoring and point-of-care [115,116,117].

**Figure 7:**
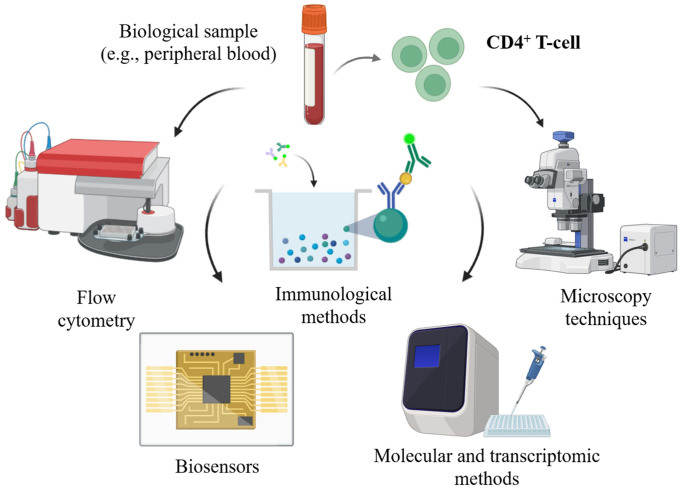
Schematic illustration of conventional and biosensing approaches used for T-cell detection. The figure summarizes representative methods currently employed for the identification, quantification, and characterization of Th-lymphocyte populations, including flow cytometry, immunological assays, microscopy-based techniques, molecular approaches, and sensing devices.

In response to the need for customization and adaptation for rapid detection, and in response to some of the most important limitations of classical methods, interest in biosensors has increased considerably in recent years. In the context where flow cytometry remains the gold standard method but is difficult to implement in conditions outside laboratories, more and more alternative platforms based on biosensors have been developed, especially integrated with microfluidics, with the aim of providing rapid, portable solutions with potential for point-of-care application. In Figure 7, we present, in schematic form, some of the main methods for detecting CD4 cells.

**Table 2:** Main methods used for T-cell detection.

Method	Principle	Provided Information	Advantages	Limitations	Applications	Ref.
Flow cytometry	fluorescent labeling of surface markers (CD3, CD4, CD8) and optical signal detection	absolute CD4 count, CD4/CD8 ratio, phenotypic profiling	high accuracy and specificity, multiparametric analysis, clinical gold standard	expensive instrumentation, need for trained personnel, low portability, static snapshot of cell populations	clinical diagnostics (HIV monitoring), immunophenotyping, oncology	[118,119,120,121,122,123]
Immunological assays (e.g., ELISA and cytokine-based)	quantification of cytokines secreted by CD4 T cells (e.g., IFN-γ, IL-4, IL-17)	indirect assessment of CD4 T-cell activation and polarization	relatively simple, useful for functional assessment	indirect measurement, lack of single-cell resolution, time-consuming, poor portability	immunological studies, inflammation and infection research	[124,125,126,127]
Microscopy and imaging techniques	visualization of CD4 marker expression and cell interactions	spatial and morphological information	high-resolution qualitative data, mechanistic insights	low throughput, difficult standardization, not suitable for routine diagnostics	fundamental immunology research	[128,129,130,131,132]
Molecular and transcriptomic methods (RT-PCR, RNA-seq)	detection of gene expression profiles associated with CD4 T-cell function	functional and transcriptional state	high sensitivity and specificity, deep biological insight	expensive, indirect, complex sample preparation, not real-time	research, disease mechanism studies	[133,134,135]
Biosensors (e.g., electrochemical)	changes in current or impedance upon CD4 cell binding	quantitative CD4 detection	high sensitivity, label-free detection, low power requirements	signal interference in complex samples, reproducibility challenges	point-of-care prototypes, screening tools	[136,137,138,139,140,141]
Microfluidic biosensor platforms	controlled manipulation of cells combined with biosensing modules	CD4-cell counting and capture	reduced sample volume, automation, integration potential	device complexity, fabrication costs, scalability issues	HIV monitoring, experimental diagnostics	[141,142,143,144,145]

#### 3.4.1. Flow Cytometry

Flow cytometry remains, without a doubt, the reference method for the detection of CD4^+^ T lymphocytes [118]. Its ability to simultaneously analyze multiple surface markers and differentiate cell subpopulations makes this technique an extremely powerful tool, both in the clinic and in research. Based on the use of fluorescent antibodies directed against CD3, CD4 and other relevant molecules, flow cytometry provides detailed information on the absolute number of CD4 T cells, the CD4/CD8 ratio and population changes [119,120,121,122,123]. This is extremely relevant, for example, in the monitoring of HIV patients or in the evaluation of the immune response in oncological therapies. However, despite its accuracy, this method is dependent on complex infrastructure, specialized personnel and fresh samples. All of this limits its use outside of well-equipped laboratories. In addition, cytometry provides a static image of cell populations, without always capturing the functional dynamics or the interactions of cells with their immediate environment.

#### 3.4.2. Immunological Methods

Classical immunological methods, such as ELISA or cytokine-based assays, partially complement this information by assessing the functional activity of Th cells [124,125,126]. By measuring the concentrations of IL-12, IFN-γ, IL-4 or IL-17, these techniques allow inferences on the activation state and functional polarization of CD4 subpopulations. However, they remain indirect methods, which do not provide information on individual cells and cannot clearly distinguish between cellular sources of cytokines in a complex biological environment. Furthermore, one can argue that these assays are generally time-consuming and difficult to integrate into miniaturized or portable systems, which reduces their attractiveness for rapid applications.

#### 3.4.3. Microscopy Techniques

Microscopy and cell imaging techniques bring another level of detail, allowing the observation of CD4 marker expression and T-cell interactions with other components of the immune system [128,129,130,131]. These methods are extremely valuable for understanding fundamental mechanisms. It is acknowledged that they are useful for investigating and researching specific aspects, but they are too difficult to standardize for quantitative analysis, and, in particular, they are not suitable for routine and rapid applications or for dynamic monitoring of cell populations in real clinical samples.

#### 3.4.4. Molecular and Transcriptomic Methods

In the same context, molecular and transcriptomic approaches (such as RT-PCR and RNA-seq) [133,134,135] provide profound, concrete and specific information about the functional state of CD4 T cells (especially correlated with various pathologies or infections), but they are expensive, indirect and far from being compatible with the idea of rapid or point-of-care detection.

#### 3.4.5. Biosensing Approaches

In recent years, increasing attention has been directed toward the development of biosensing platforms for CD4^+^ T-cell detection, particularly in the context of rapid diagnostics and POC applications. These sensing approaches rely on different transduction mechanisms, including electrochemical, optical, and microfluidic detection strategies, aiming to improve portability, assay speed, and integration into miniaturized systems.

From a technological point of view, T lymphocytes have several characteristics that make them attractive for biosensing applications [139,146,147]. One of them is the relatively uniform size and shape of the cells [148,149], which may facilitate their manipulation and capture in microfluidic systems or on functionalized surfaces. The second is the composition of the cell membrane and the expression of specific surface markers, (CD3, CD4 or CD8) [150]; this allows the development of recognition ligands, including antibodies, aptamers or synthetic recognition molecules, that can be used for the selective capture of these cells. Furthermore, T lymphocytes are present in accessible biological fluids (such as peripheral blood) [151]; this facilitates their integration into diagnostic platforms. These biological characteristics, combined with the relative accessibility of samples, place T lymphocytes at the interface between immunology and detection technologies, providing a solid basis for the development of systems that can quantify and possibly monitor changes in the immune system in real time.

*Sensing platforms for the dynamic behavior of T lymphocytes*: An important aspect related to the development of detection platforms, which is worth mentioning, is the dynamic behavior of T lymphocytes in the peripheral circulation and in lymphoid tissues [152]. Their concentration, activation state and expression of surface markers can change rapidly depending on infections, inflammation or therapeutic treatments. T lymphocytes are not “static targets” but are dynamic biological entities whose activity and function adapt to the environmental biological conditions [50,153,154]. This dynamic behavior implies that detection strategies relying on static capture may fail to reflect the true functional state of T cells. Therefore, any detection technology must take into account both the quantitative variations and the functional heterogeneity of these cells.

An example in this regard is the study by Bousso and Robey [152], which shows that T lymphocytes are not activated by static or prolonged contact, but rather, by a deeply dynamic process. They demonstrated that T cells migrate continuously and rapidly through the lymph nodes, coming into successive contact with antigen-presenting cells. These initial contacts are brief and transient, and their role is to “scan” the environment, not to become immediately activated. The T cell quickly assesses whether the presented antigen is relevant, without stopping to migrate. T-cell activation occurs only when the antigenic signal exceeds a critical threshold, both in terms of TCR receptor affinity and the duration of contact.

Another example is provided by the study of Meng et al. [141] in which the authors discuss that microfluidic assays, which are systems that combine fluid handling microchannels with detection modules, are among the most studied for counting CD4^+^ lymphocytes and assessing immunological status; this is particularly suitable for HIV and other infectious diseases.

*Electrochemical biosensors*: Developments in biosensors are not limited to just one type of transduction. Electrochemical biosensors, for example, combine the advantages of high sensitivity and rapid response with the possibility of using selective biological components such as antibodies or other synthetic receptors. In their study, Cui et al. [140] highlight the consistent progress in electrochemical biosensors for disease diagnosis, highlighting rapid responses, sensitivity and low operating costs, but also mention major challenges, such as the variability of results in complex biological matrices and the need for extensive clinical validation before use in real practice.

A concrete and very recent example is an electrochemical biosensor integrated into a microfluidic device, developed for the detection of CD4^+^ T cells in blood samples, using electrochemical impedance spectroscopy as the transduction principle [136]. This platform combines the functionalization with anti-CD4 antibodies directly on an electrode integrated into a microfluidic channel. It allows cell detection in a clinically relevant linear range, with detection limits and sensitivity adequate for differentiating between normal and reduced levels of CD4^+^ T cells in HIV-positive patients [136]. This offers a relatively simple method of transduction, without the need for fluorescent markers or bulky equipment, which makes it attractive for point-of-care applications and screening in resource-limited settings.

*Optical biosensors:* Compared to classical methods, which require complex equipment and high costs, optical biosensors open avenues for more rapid, simple and possibly adaptable devices that are suitable for point-of-care applications and that also have significant practical value. Thus, in their study, Salvo et al. [139] highlighted how optical biosensors can change CD4 T-cell detection in a rapid and more accessible manner using specific interactions between antibodies and/or cellular markers. For instance, such a detection method would include fluorescently labeling major histocompatibility complex proteins loaded with antigen-derived peptides [155]. Another example is counting CD4+ cells in the blood using a combination of antibodies conjugated with fluorescein isothiocyanate [156].

Thus, by using varied surfaces biofunctionalized with anti-CD4 antibodies, aptamers or other recognition molecules, such sensing platforms can allow for the selective capture of CD4 T cells. Moreover, the major advantage of these systems lies in their potential for miniaturization and integration into portable devices, as well as in the possibility of real-time analysis.

#### 3.4.6. Microfluidics Assays

This trend towards integrating biosensors with microfluidics is not isolated. Microfluidic assays for CD4-cell counting are increasingly being addressed in the literature, precisely because they allow the manipulation of biological samples in a controlled and automated manner, reducing manual handling and the risk of human error [141,143,144,145,157,158]. Such microfluidic systems, although still largely in the research stage, offer a way to overcome some of the main barriers of classical methods: cost, complexity and the need for laboratory infrastructure.

Despite this, there are conflicting opinions regarding the current utility of biosensors in CD4 lymphocyte detection. While some researchers emphasize the potential advantages of microfluidic assays and electrochemical biosensors for the global health context, others point out that most of the systems developed so far are still tested on model samples or simplified systems, and their performance in whole blood samples remains insufficiently demonstrated. This, of course, raises questions about the reproducibility, specificity, and robustness of these biosensors under variable clinical conditions, where serum proteins, non-target cells, or other interfering factors can generate false signals or reduce the accuracy of measurements. Moreover, the complete integration of cell separation, functionalization, and detection in a single device, without manual steps, still remains a significant technological challenge.

Furthermore, although electrochemical and microfluidic biosensors for CD4^+^ T cells are among the most studied, there is still a gap between published research and actual clinical implementation; most of the work is at the proof-of-concept or experimental optimization level, without validation in large clinical sample sets. Hence, we can admit the need for robust testing strategies, standardization, and adaptation to regulatory and practical requirements before these technologies can be widely adopted.

In order to provide a clearer comparative overview of currently available CD4^+^ T-cell detection and sensing approaches, a structured comparison of representative methods was included. In this regard, we refer to Table 3.

### 3.5. Potential Use of Magnetotactic Bacteria for Detecting CD4 T Cells

The analysis of current methods for detecting CD4^+^ T lymphocytes has revealed constant technological progress but also raises a series of recurring limitations that persist, regardless of the platform used. Whether we are talking about flow cytometry, electrochemical biosensors or integrated microfluidic systems, most approaches rely on multiple sample processing steps, complex surface functionalization and relatively sophisticated experimental infrastructure. These aspects become even more problematic when detection is performed in complex biological environments, where nonspecific interference, cell losses and sample variability can significantly affect the accuracy of the results.

Based on these observations, it becomes evident that alternative or complementary directions should be explored, allowing for a more dynamic interaction between the detection system and the target cells. In this sense, the use of magnetotactic bacteria opens a different perspective on the biosensor concept, shifting the focus from static devices to active biological systems capable of responding to well-controlled physical stimuli. Their intrinsic magnetic properties, precise organization of magnetosomes and ability to orient in a magnetic field offer a set of functionalities that are not accessible by conventional biosensors.

Moreover, MTBs can be viewed not only as transduction elements but also as multifunctional biological platforms, capable of combining biological recognition, targeted manipulation and signal amplification in a single system. This approach reconsiders the way in which immune cell detection, especially of CD4 populations, is performed, with the premises for strategies that are simpler technologically but more biologically adaptive. Thus, the transition to the use of MTBs does not represent an abandonment of the progress made to date but a natural conceptual extension, oriented towards overcoming current limitations and towards exploring detection mechanisms directly inspired by biology.

#### 3.5.1. Why Use Magnetotactic Bacteria in the Detection of Th Cells?

**Figure 8:**
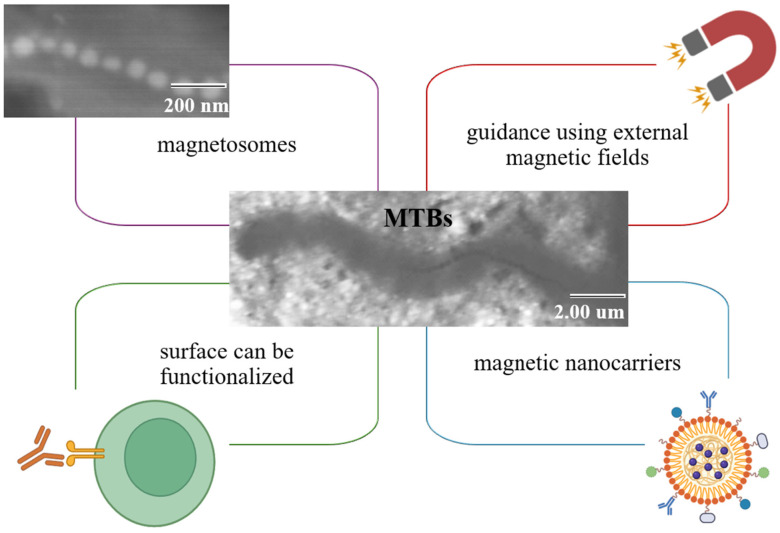
Distinctive features of magnetotactic bacteria that make them promising for detection of T cells.

Once we have identified the limitations of classical methods for detecting CD4^+^ T lymphocytes and the challenges faced by conventional biosensors, it may seem natural to ask whether there are biological elements that can be used as an active part of a detection platform. In this search, magnetotactic bacteria appear as a coherent potential solution, motivated by unique properties that clearly differentiate them from synthetic materials commonly used in biosensors [19,21] (see Figure 8).

In essence, MTBs are microorganisms that synthesize magnetic crystalline structures, called magnetosomes, organized in internal chains. These magnetosomes give the bacteria a net magnetic moment, which allows them to orient and move according to the direction of an external magnetic field [159]. This externally controlled magnetotaxis gives them a functional advantage over synthetic magnetic materials, with promising applications and great technological complexity [19]. In practice, MTBs can be guided and manipulated using external magnetic fields, making them suitable as dynamic elements in a cellular detection system.

In addition to this targeted manipulation, magnetosomes themselves present some differences between species, both in size (35 to 120 nm) and shape (cuboctahedral, elongated prismatic or bullet-shape), but within the same species, they are relatively homogeneous magnetic nanoparticles (in size and shape) with well-defined magnetic properties [160,161]. This homogeneity is difficult to achieve by synthesizing artificial magnetic materials and, in many cases, requires complex fabrication and purification steps [20,21]. In contrast, MTBs synthesize magnetosomes biologically, with an internal organization adapted to their environment and with a structural consistency that only biology can effectively reproduce. This biological organization of magnetosomes provides well-defined magnetic properties together with a structurally organized “platform” that may support their integration into adaptable cell detection or separation systems.

In addition to their manipulation and magnetic properties, MTBs present another important advantage: their cell surface can be biochemically functionalized to recognize target cells [162,163,164,165], including CD4^+^ T lymphocytes. This means that, using specific antibodies or other recognition molecules, MTBs can act as a selective capture element directly in a biological sample flow. This strategy may, to some extent, overcome the limitations of conventional biosensors, where the biofunctionalization of the sensor surface often remains a critical challenge, susceptible to loss of activity, degradation or interference from the biological environment. Although MTB-based systems also require surface functionalization, the natural membrane structure of magnetosomes may provide a biologically compatible interface for biomolecule immobilization and targeted cell interaction. Nevertheless, challenges related to the reproducibility, biological heterogeneity, and standardization of functionalization strategies still need to be addressed before broader diagnostic implementation.

MTBs are not just “passive” entities that can be used as magnetic nanocarriers, but rather, they can be integrated into a hybrid system in which, for example, magnetic manipulation, selective capture and signal transduction are directly correlated, without requiring multiple processing steps. As a hypothetical example, the application of a controlled variation of the magnetic field can cause the movement of functionalized bacteria towards CD4^+^ cells and thus amplify the interaction and increase the detection signal. This hybrid approach, combining active biological elements with external physical manipulation, may offer a way out of the dilemma of traditional biosensors, where the signal is often weak, unclear and influenced by unwanted interference.

The arguments for using MTBs in CD4^+^ T-cell detection are not limited to the physical or functional properties of bacteria but also extend to their potential for integration into a fully self-organizing and adaptive system. Instead of the detector being a passive object, inherently rigid in design, MTBs can offer a component of biological autonomy, responding to stimuli provided by the environment and the external magnetic system in a way that is not possible with standard synthetic materials. This perspective not only addresses some of the limitations previously identified in conventional biosensors but also opens up the opportunity to develop cellular detection platforms that are closer to real biological processes, with a more natural integration between molecular recognition and the physical response of the system.

Thus, the rationale for using magnetotactic bacteria in the detection of CD4^+^ T lymphocytes lies not only in a simple substitution of magnetic materials but in a redefinition of how cell detection can be achieved: moving from passive devices to active biological systems, where recognition, manipulation, and signaling are intrinsically interconnected. This approach can provide not only performance gains but also superior adaptability, paving the way for innovative, robust, and useful biomedical applications in immunological diagnostics.

#### 3.5.2. How Can Magnetotactic Bacteria Improve the Detection of CD4^+^ T Cells?

**Figure 9:**
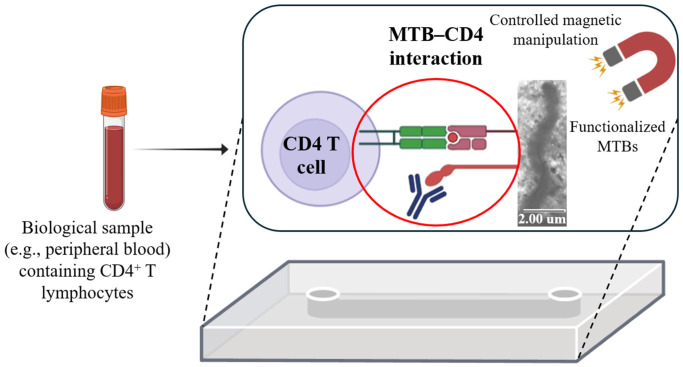
Conceptual design for CD4 T-cell detection device using MTBs. The schematic illustrates the potential integration of magnetotactic bacteria (MTBs) with selective surface functionalization for magnetic manipulation and target-cell recognition. Such an approach is proposed as an exploratory strategy for future adaptable and miniaturized biosensing applications.

To fully understand the potential of magnetotactic bacteria in detecting CD4^+^ T lymphocytes, it is useful to imagine how they would behave in a hybrid system in which biological and physical elements work together. Unlike conventional biosensors, which sometimes treat the biological signal as a “passive” phenomenon that must be transposed into an optical or electrical signal, MTBs can be used as active actors in the process of recognizing, capturing, manipulating and amplifying the detection signal. In Figure 9, we present a conceptual design of a device using MTBs.

The first distinctive element is the ability of MTBs to be guided by an external magnetic field. The magnetosomes synthesized by these bacteria generate an internal magnetic moment, which allows them to respond to variations in the magnetic field with remarkable precision. In a cellular detection system, this can be exploited to direct the manipulation of functionalized bacteria to the area where CD4^+^ lymphocytes are located, thus minimizing sample losses and increasing the probability of specific interaction. This guided manipulation could not only concentrate bacteria in the vicinity of target cells but could also generate a direct physical signal (quantifiable magnetic changes) when the interaction occurs. At the same time, the biological surface of MTBs can be biofunctionalized with recognition molecules, such as anti-CD4 antibodies, aptamers, or protein fragments with an affinity for T-helper-cell markers. This functionalization would give the bacteria additional biological specificity, transforming them from natural magnetic structures into cellular recognition platforms. When a functionalized MTB would bind to a CD4 expressed on the surface of a T cell, the biological interaction should be naturally selective, thus reducing nonspecific interference (one of the main limitations of standard electrical or optical biosensors).

Another mechanism by which MTBs could amplify the detection signal lies in their collective magnetizable effect [166,167]. Unlike dispersed synthetic magnetic particles, magnetosomes inside bacteria are arranged in a well-defined and oriented internal structure [168], which can lead to a stronger and more predictable magnetic response when exposed to an external magnetic field. This property can be exploited in a system where the magnetic signal generated or modified by the interaction of the bacterium with a CD4 cell can be directly detectable by sensitive magnetic methods (e.g., magnetometers), providing an alternative and complementary transduction pathway to optical or electrical ones.

On top of that, MTBs are living biological entities, capable in some contexts of responding to environmental stimuli or modifying their biochemical interactions depending on local conditions [169,170]. This biological dynamic differentiates them from passive synthetic materials and opens the possibility of designing detection systems that do not just read a signal but interrogate the behavior of target cells in real time. In such scenarios, interactions between MTBs and CD4^+^ lymphocytes could provide information not only about the presence or absence of the cells but also about the functional states they express, which would provide a significant advantage over systems that are limited to numerical quantification.

A final important mechanism is the possibility of combining magnetic manipulation with sequential signal amplification. By controlled application of magnetic field variations (e.g., oscillatory or pulsating), repeated changes in the position and orientation of the functionalized bacteria could be induced. This repeated manipulation could function as an active “scanning” process of the sample, which could increase the efficiency of capture.

Instead of treating biological recognition and physical transduction as separate elements, MTBs offer a natural integration between biology and physics, which may lead to more robust, sensitive, and adaptable detection systems than those developed so far.

In this context, the transition from conventional biosensors to MTB-based approaches is not only a change in technology but also a paradigm shift: from passive systems that wait for a biological signal to active systems that participate in and shape biological interaction to produce detectable and clinically meaningful signals.

An important aspect to mention in this regard is that the concept of magnetic-based T-cell isolation is not novel. Synthetic magnetic beads are already widely used in commercially available immunoseparation kits for CD4^+^ T-cell enrichment. These systems are well-established, highly standardized, and suitable for routine laboratory applications. In this context, MTB-based approaches should not be considered direct replacements of existing magnetic bead technologies, but rather, as alternative bioinspired systems with distinct characteristics. However, MTB-based systems remain at an early conceptual stage and require significant advances in control, standardization, and reproducibility before any comparison with established commercial technologies can be made.

## 4. Discussion

When examining all methods for detecting CD4^+^ T cells, the technological advances made in recent years are impressive. Flow cytometry, for example, is the gold standard, but it is hard to ignore the fact that it remains a laboratory tool. It is bulky, expensive, dependent on trained personnel and very unsuitable for point-of-care applications, which are often extremely necessary. There is a factual point: the literature makes it quite clear that microfluidic assays and other alternative approaches were developed precisely to solve the problem of cost and accessibility [141,143,171].

On the other hand, biosensors and microfluidic-integrated platforms have demonstrated the potential to provide more accessible and dynamic alternatives, with the possibility of real-time detection and in a portable format, which is evident from the studies dedicated to these technologies. For instance, plasmonic biosensors based on LSPR or SERS have been explored for the detection of biomolecules, surface antigens, and even immune cells [139,156,172,173,174]. They offer high sensitivity due to the label-free sensing approach and have great potential for real-time cellular analysis. Despite this, direct implementation for targeting CD4^+^ T lymphocytes is limited.

The current systems focus on closely related immune markers or rely on model cell systems, suggesting that plasmonic detection of CD4^+^ T cells is technically feasible but still underexplored.

Some of the solutions proposed in the literature are still at the proof-of-concept stage, firstly tested in environment models or with simplified samples, while their performance when using real clinical samples is often insufficiently demonstrated.

This highlights the need for rigorous validation, reproducibility and adaptation to real clinical contexts before they could be widely adopted. At the same time, advances in biosensor design suggest that the field is in an accelerated maturation phase.

Given these challenges and opportunities, the use of active biological elements, such as magnetotactic bacteria, is emerging as a highly promising research direction. Their natural magnetic properties and ability to be functionalized for selective recognition provide the conceptual framework for systems that combine biological recognition with controlled physical manipulation. This approach could overcome some of the limitations of the current approaches and may enable the development of more dynamic and adaptable sensing platforms.

The proposal of magnetotactic bacteria as a central element in a possible CD4^+^ T-lymphocyte detection system does not stem from the desire to replace established methods, but rather, from the need to explore an area that remains relatively underexploited in immunodiagnostics (the use of active biological systems, not just as a passive support but as a functional part of the detection mechanism). In the recent literature, MTBs are predominantly studied for the magnetic properties of magnetosomes or for applications in nanomedicine, drug delivery and imaging [175,176,177,178] but are much less often studied in contexts of targeted cellular detection [178,179,180], which, paradoxically, represents both a limitation and a clear opportunity.

A comparative analysis of CD4^+^ T-cell detection strategies highlights differences between established technologies and emerging approaches. Flow cytometry remains the gold standard due to its high sensitivity, quantitative capability, and ability to perform multiparametric immunophenotyping. Microscopy-based techniques provide spatial resolution but are limited in throughput and quantitative analysis. Biosensor platforms, including electrochemical and microfluidic systems, offer advantages in terms of miniaturization and rapid detection, although they often face challenges related to biofunctionalization stability and matrix interference. In contrast, magnetotactic bacteria-based systems are still at an early developmental, or proof-of-concept, stage. They offer a unique bioinspired alternative characterized by intrinsic magnetic responsiveness and the potential for simplified cell capture.

MTB-based approaches currently cannot match the multiparametric analysis and detailed capabilities of flow cytometry or advanced microscopy-based techniques. Nevertheless, they may offer advantages for the development of simplified, miniaturized, and portable biosensing platforms. Magnetosome-based systems could support rapid cell detection, magnetic enrichment, and POC applications outside specialized laboratory settings. However, further studies are required for the development and standardization, reproducibility, and clinical validation of these approaches.

An important argument in favor of MTBs is the fact that they combine in a single entity several functions that, in conventional biosensors, are distributed across different components: magnetic element, biofunctionalization support and mobile unit. This natural integration reduces the conceptual complexity of the platform and, at least theoretically, can diminish signal losses associated with multiple processing, washing or amplification steps. Of course, this proposal is not without risks or unknowns, and the current literature does not yet provide sufficient experimental data to validate the use of MTBs in real clinical trials. However, it is precisely this gap that justifies incorporating this concept into an exploratory framework. The proposal to use MTBs should not be considered as an immediate alternative to flow cytometry or classical biosensors, but rather, as an interesting attempt to complete the understanding of how immune cell detection could evolve while integrating active biological systems and more closely replicating the complexity of the real biological environment.

From an outlook perspective, CD4^+^ T-lymphocyte analysis is expected to evolve toward more rapid, minimally invasive, and highly integrated detection strategies. While conventional methods remain the gold standard for immunophenotyping, future developments are likely to focus on simplified, portable, and point-of-care-compatible platforms capable of operating in complex biological environments. In this context, emerging biosensing technologies, including microfluidic and electrochemical systems, as well as bioinspired approaches based on MTBs, may contribute to the development of next-generation diagnostic tools.

## 5. Conclusions

This review stresses the central role of CD4^+^ T lymphocytes in orchestrating the immune response and their major clinical importance in numerous pathologies. The analysis of current methods for detecting CD4^+^ cells showed that, although techniques such as flow cytometry or biosensor-based platforms offer high performance under controlled conditions, they remain limited by technological complexity, costs, the need for specialized infrastructure and, last but not least, the difficulty of integration into a simple and rapid analysis flow. In this context, the proposal to use magnetotactic bacteria appears to be an alternative conceptual direction, which brings together in a single entity well-defined magnetic properties, biocompatibility and the ability to be actively manipulated under an external magnetic field. MTBs should not be seen as a direct substitute for established methods but as an active biological element that can open new perspectives in the development of more flexible detection platforms closer to natural biological processes. The application of these systems for the specific detection of CD4^+^ T cells remains largely at the proof-of-concept stage, requiring further validation regarding reproducibility, standardization, and clinical applicability.

Taking everything into account, this review does not propose a definitive solution, but rather, it outlines an emerging research direction, located at the intersection of immunology, nanobiotechnology and bioengineering. The main value of the analyzed concept lies in its exploratory potential and in its ability to stimulate the development of detection systems that go beyond the passive sensor paradigm, opening the way to hybrid, adaptive and biologically relevant platforms for future applications in immunological diagnostics.

## Figures and Tables

**Table 1:** Main subpopulations of T lymphocytes.

T Cell	Main Cytokine/ Effector Molecules	Main Role	Biological Context	Ref.
CD8^+^ T cells	IFN-γ, TNF-α, perforin, granzymes	direct killing of infected or malignant cells	viral infections, tumor surveillance, antitumor immunity	[47,48,49]
Th1 cells (CD4^+^)	IFN-γ, IL-2, TNF-α, TNF-β	activation of macrophages and support of cell-mediated immunity	intracellular bacterial and viral infections	[41,51,61,67]
Th2 cells (CD4^+^)	IL-4, IL-5, IL-6, IL-10, IL-13	B-cell activation and IgE production	parasitic infections, allergic diseases, asthma	[53,54,55,56,61,67]
Th17 cells (CD4^+^)	IL-17, IL-17F, IL-22	induction of inflammation and neutrophil recruitment	extracellular bacterial and fungal infections, autoimmunity	[57,58,59,61,68]
Regulatory T cells (Tregs, CD4^+^)	IL-10, TGF-β	suppression of immune responses and maintenance of tolerance	prevention of autoimmunity, control of chronic inflammation	[41,64,65,66]

**Table 3:** Comparative analytical performance of representative CD4^+^ T-cell detection methods (based on Refs. [118,119,120,121,122,123,124,125,126,127,128,129,130,131,132,133,134,135,136,137,138,139,140,141,142,143,144,145,157,158]).

Method	Sample Type	Analytical Performance	Assay Time	Portability	Validation Status	Performance in Complex Samples
Flow cytometry	whole blood	high accuracy, multiparametric CD4/CD8 quantification	~1–3 h	low	fully validated	high
ELISA/cytokine assays	serum/plasma	high sensitivity (indirect CD4 functional assessment)	>2 h	low	validated	low–moderate
Microscopy-based methods	fixed cells	high single-cell specificity	several hours	low	validated (research use)	low–moderate
Molecular methods (e.g., RT-PCR, RNA-seq)	whole blood	high sensitivity (CD4^+^/Th cell gene expression profiling)	several hours–days	low	validated (research/clinical)	moderate
Electrochemical biosensors	whole blood/ serum	high sensitivity	<1 h	high	emerging/partially validated	moderate
Microfluidic biosensors	whole blood	high sensitivity	<1 h	high	partially validated	good
MTB-based systems (conceptual)	proposed for blood samples	not yet standardized	experimental	potentially high	exploratory concept	not yet demonstrated

## Data Availability

Data are contained within the article.

## Supplementary Materials

The following supporting information can be downloaded at: https://www.mdpi.com/article/10.3390/s26113324/s1, Table S1: PRISMA checklist [181].
